# Supplementary material for: Biogeography and environmental conditions shape bacteriophage-bacteria networks across the human microbiome
Source: PLoS Comput Biol. 2018 Apr 18;14(4):e1006099. doi: 10.1371/journal.pcbi.1006099 (PMC5927471; doi:10.1371/journal.pcbi.1006099)
Supplement: S1 Table — (PDF) [file pcbi.1006099.s015.pdf]

Table S1

Study	Citation	Virome Quality Control Measures
Diet & the Gut Virome	Minot, 2011	• 16S rRNA gene qPCR revealed reduction in bacterial DNA of at least 10,000X.<br>• Alignment of shotgun sequences revealed 35X reduction in 16S rRNA gene alignments in virome compared to bacteria shotgun.<br>• Electron microscopy and nucleic acid stain techniques visually confirmed lack of bacteria in virome samples.
Skin Virome	Hannigan, 2015	• Significant reduction in reads mapping to 16S rRNA gene sequence, compared to bacteria shotgun dataset.<br>• Significant reduction in reads mapping to human genome, compared to bacteria shotgun dataset.<br>• Average viral relative abundance of 0.4% in bacterial shotgun dataset.
Twin Gut Virome	Reyes, 2010	• Confirmation that 2.5% of bacterial shotgun reads mapped to virome, and 76% of virome reads matched the shotgun 2.5%.
