# Supplementary material for: Biogeography and environmental conditions shape bacteriophage-bacteria networks across the human microbiome
Source: PLoS Comput Biol. 2018 Apr 18;14(4):e1006099. doi: 10.1371/journal.pcbi.1006099 (PMC5927471; doi:10.1371/journal.pcbi.1006099)
Supplement: S2 Table — Citation sources are also included. (PDF) [file pcbi.1006099.s016.pdf]

Table S2

Bacterial Host	Bacteriophage	Interaction	Citation
Achromobacter xylosoxidans	Pbunalikevirus phiFenriz	Negative	Malki, *et al.* 2015.
Achromobacter xylosoxidans	Pbunalikevirus phiHabibi	Negative	Malki, *et al.* 2015.
Achromobacter xylosoxidans	Pbunalikevirus phiMoody	Negative	Malki, *et al.* 2015.
Achromobacter xylosoxidans	Pbunalikevirus phiVader	Negative	Malki, *et al.* 2015.
Acinetobacter baumannii	Pseudomonas phage PA1phi	Negative	Kim *et al.* 2012.
Acinetobacter baumannii	Acinetobacter phage Petty	Positive	Edwards *et al.* 2015.
Arthrobacter sp.	Pbunalikevirus phiHabibi	Positive	Malki, *et al.* 2015.
Arthrobacter sp.	Pbunalikevirus phiVader	Positive	Malki, *et al.* 2015.
Arthrobacter sp.	Pbunalikevirus phiMoody	Positive	Malki, *et al.* 2015.
Arthrobacter sp.	Pbunalikevirus phiFenriz	Positive	Malki, *et al.* 2015.
Arthrobacter sulfonivorans	Pbunalikevirus phiHabibi	Positive	Malki, *et al.* 2015.
Arthrobacter sulfonivorans	Pbunalikevirus phiVader	Positive	Malki, *et al.* 2015.
Arthrobacter sulfonivorans	Pbunalikevirus phiMoody	Positive	Malki, *et al.* 2015.
Arthrobacter sulfonivorans	Pbunalikevirus phiFenriz	Positive	Malki, *et al.* 2015.
Bacillus pumilus	Enterobacteria phage phi92	Negative	Schwarzer, *et al.* 2012.
Bacillus pumilus	Enterobacteria phage K1F	Negative	Schwarzer, *et al.* 2012.
Bacillus amyloliquefaciens	Enterobacteria phage phi92	Negative	Schwarzer, *et al.* 2012.
Bacillus amyloliquefaciens	Enterobacteria phage K1F	Negative	Schwarzer, *et al.* 2012.
Bacillus subtilis	Enterobacteria phage phi92	Negative	Schwarzer, *et al.* 2012.
Bacillus subtilis	Enterobacteria phage K1F	Negative	Schwarzer, *et al.* 2012.

Bacterial Host	Bacteriophage	Interaction	Citation
Burkholderia cenocepacia	Burkholderia phage KL1	Positive	Hargreaves, *et al.* 2014.
Burkholderia cenocepacia	Burkholderia phage KS14	Positive	Edwards *et al.* 2015.
Chryseobacterium sp.	Pbunalikevirus phiHabibi	Positive	Malki, *et al.* 2015.
Chryseobacterium sp.	Pbunalikevirus phiFenriz	Positive	Malki, *et al.* 2015.
Chryseobacterium sp.	Pbunalikevirus phiMoody	Positive	Malki, *et al.* 2015.
Chryseobacterium sp.	Pbunalikevirus phiVader	Positive	Malki, *et al.* 2015.
Clostridium perfringens	Clostridium phage phiCP7R	Positive	Edwards *et al.* 2015.
Clostridium perfringens	Clostridium phage phiZP2	Positive	Edwards *et al.* 2015.
Corynebacterium glutamicum	Corynebacterium phage P1201	Positive	Edwards *et al.* 2015.
Cronobacter sakazakii	Cronobacter phage CR5	Positive	Edwards *et al.* 2015.
Escherichia coli	Pbunalikevirus phiHabibi	Positive	Malki, *et al.* 2015.
Escherichia coli	Pbunalikevirus phiMoody	Positive	Malki, *et al.* 2015.
Escherichia coli	Pbunalikevirus phiFenriz	Positive	Malki, *et al.* 2015.
Escherichia coli	Pbunalikevirus phiVader	Positive	Malki, *et al.* 2015.
Escherichia coli	Pseudomonas aeruginosa phage F116	Negative	Jensen *et al.* 1998.
Escherichia coli	Pseudomonas phage D3	Negative	Jensen *et al.* 1998.
Enterobacter aerogenes	Pseudomonas phage PA1phi	Negative	Kim *et al.* 2012.
Erwinia amylovora	Erwinia phage phiEaH2	Positive	Edwards *et al.* 2015.
Escherichia coli	Pseudomonas phage PA1phi	Negative	Kim *et al.* 2012.
Escherichia coli	Enterobacteria phage K1F	Positive	Schwarzer, *et al.* 2012.
Escherichia coli	Enterobacteria phage phi92	Positive	Schwarzer, *et al.* 2012.
Klebsiella pneumoniae	Klebsiella phage KP15	Positive	Edwards *et al.* 2015.
Lactococcus lactis	Lactococcus phage 949	Positive	Edwards *et al.* 2015.
Lactococcus lactis	Lactococcus phage phiL47	Positive	Edwards *et al.* 2015.
Listeria monocytogenes	Pseudomonas phage PA1phi	Positive	Kim *et al.* 2012.
Microbacterium sp.	Pbunalikevirus phiVader	Positive	Malki, *et al.* 2015.
Microbacterium sp.	Pbunalikevirus phiMoody	Positive	Malki, *et al.* 2015.

Bacterial Host	Bacteriophage	Interaction	Citation
Microbacterium sp.	Pbunalikevirus phiHabibi	Positive	Malki, *et al.* 2015.
Microbacterium sp.	Pbunalikevirus phiFenriz	Positive	Malki, *et al.* 2015.
Mycobacterium smegmatis	Mycobacterium phage SWU1	Positive	Edwards *et al.* 2015.
Pseudomonas aeruginosa	Pbunalikevirus phiMoody	Positive	Malki, *et al.* 2015.
Pseudomonas aeruginosa	Pbunalikevirus phiVader	Positive	Malki, *et al.* 2015.
Pseudomonas aeruginosa	Pbunalikevirus phiFenriz	Positive	Malki, *et al.* 2015.
Pseudomonas aeruginosa	Pbunalikevirus phiHabibi	Positive	Malki, *et al.* 2015.
Pseudomonas aeruginosa	Pseudomonas aeruginosa phage F116	Positive	Jensen *et al.* 1998.
Pseudomonas aeruginosa	Pseudomonas phage D3	Positive	Jensen *et al.* 1998.
Photobacterium angustum	Vibrio phage KVP40	Negative	Matsuza *et al.* 1992.
Photobacterium leiognathi	Vibrio phage KVP40	Positive	Matsuza *et al.* 1992.
Propionibacterium acnes	Propionibacterium phage PHL112N00	Positive	Edwards *et al.* 2015.
Pseudomonas aeruginosa	Pseudomonas phage PA1phi	Positive	Kim *et al.* 2012.
Pseudomonas putida	Enterobacteria phage K1F	Negative	Schwarzer, *et al.* 2012.
Pseudomonas putida	Enterobacteria phage phi92	Negative	Schwarzer, *et al.* 2012.
Ralstonia solanacearum	Ralstonia phage RSM3	Positive	Edwards *et al.* 2015.
Ralstonia solanacearum	Ralstonia phage RSS1	Positive	Edwards *et al.* 2015.
Sphaerotilus natans	Pseudomonas aeruginosa phage F116	Positive	Jensen *et al.* 1998.
Sphaerotilus natans	Pseudomonas phage D3	Positive	Jensen *et al.* 1998.
Salmonella enterica	Pbunalikevirus phiFenriz	Negative	Malki, *et al.* 2015.
Salmonella enterica	Pbunalikevirus phiHabibi	Negative	Malki, *et al.* 2015.
Salmonella enterica	Salmonella phage FSL SP-058	Positive	Edwards *et al.* 2015.
Salmonella enterica	Pbunalikevirus phiVader	Negative	Malki, *et al.* 2015.
Salmonella enterica	Pbunalikevirus phiMoody	Negative	Malki, *et al.* 2015.
Salmonella enterica	Enterobacteria phage phi92	Positive	Schwarzer, *et al.* 2012.

Bacterial Host	Bacteriophage	Interaction	Citation
Salmonella enterica	Enterobacteria phage K1F	Negative	Schwarzer, *et al.* 2012.
Serratia marcescens	Pseudomonas phage PA1phi	Negative	Kim *et al.* 2012.
Shigella boydii	Pbunalikevirus phiVader	Negative	Malki, *et al.* 2015.
Shigella boydii	Pbunalikevirus phiHabibi	Negative	Malki, *et al.* 2015.
Shigella boydii	Pbunalikevirus phiFenriz	Negative	Malki, *et al.* 2015.
Shigella boydii	Pbunalikevirus phiMoody	Negative	Malki, *et al.* 2015.
Shigella flexneri	Pbunalikevirus phiFenriz	Negative	Malki, *et al.* 2015.
Shigella flexneri	Pbunalikevirus phiVader	Negative	Malki, *et al.* 2015.
Shigella flexneri	Pbunalikevirus phiMoody	Negative	Malki, *et al.* 2015.
Shigella flexneri	Pbunalikevirus phiHabibi	Negative	Malki, *et al.* 2015.
Shigella sonnei	Pseudomonas phage PA1phi	Positive	Kim *et al.* 2012.
Staphylococcus aureus	Pseudomonas phage PA1phi	Positive	Kim *et al.* 2012.
Staphylococcus epidermidis	Staphylococcus phage CNPH82	Positive	Edwards *et al.* 2015.
Staphylococcus epidermidis	Pseudomonas phage PA1phi	Negative	Kim *et al.* 2012.
Streptococcus agalactiae	Pseudomonas phage PA1phi	Negative	Kim *et al.* 2012.
Streptococcus gordonii	Pseudomonas phage PA1phi	Negative	Kim *et al.* 2012.
Streptococcus pneumoniae	Streptococcus phage K13	Positive	Edwards *et al.* 2015.
Streptococcus pneumoniae	Pseudomonas phage PA1phi	Negative	Kim *et al.* 2012.
Streptococcus salivarius	Pseudomonas phage PA1phi	Positive	Kim *et al.* 2012.
Thermus thermophilus	Thermus phage TMA	Positive	Edwards *et al.* 2015.
Vibrio natriegens	Vibrio phage KVP40	Positive	Matsuza *et al.* 1992.
Vibrio parahaemolyticus	Vibrio phage KVP40	Positive	Matsuza *et al.* 1992.
Vibrio vulnificus	Vibrio phage KVP40	Negative	Matsuza *et al.* 1992.
Xylella fastidiosa	Xylella phage Xfas53	Positive	Edwards *et al.* 2015.
